# 475. "The Outcomes and Adverse Effects of COVID-19 Therapies in High-Risk Children"

**DOI:** 10.1093/ofid/ofad500.545

**Published:** 2023-11-27

**Authors:** Johanna Holschier, Alison Boast, Christine Plover, Suzanne Boyce, Sarah McNab, Nigel Curtis, Amanda Gwee

**Affiliations:** Royal Children's Hospital Melbourne, Melbourne, Victoria, Australia; Royal Children's Hospital Melbourne, Melbourne, Victoria, Australia; The Royal Childrens Hospital, Melbourne, Victoria, Australia; The Royal Childrens Hospital, Melbourne, Victoria, Australia; The Royal Childrens Hospital, Melbourne, Victoria, Australia; The Royal Childrens Hospital, Melbourne, Victoria, Australia; The Royal Children's Hospital, Melbourne, Victoria, Parkville, Victoria, Australia

## Abstract

**Background:**

Numerous international studies have focused on the outcomes of COVID-19 therapies in adults, however data are limited on the outcomes for high-risk paediatric patients treated with similar agents.

**Methods:**

Data was collected retrospectively for high-risk children treated for symptomatic COVID-19 at a tertiary paediatric hospital in Australia, over a 12-month period (September 2021- August 2022). Indications for COVID-19 therapies are outlined in [Figure 1]; with the guideline being implemented in September, 2021.

**Figure 1:**
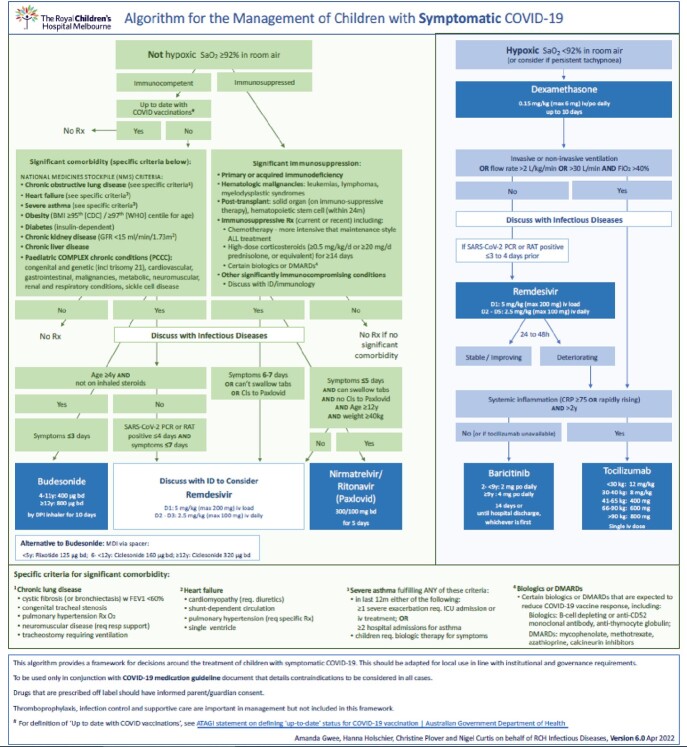
COVID-19 Treatment Algorithm

Note: Casirivimab-imdevimab & sotrovimab were included in previous iterations of the COVID-19 algorithm. Due to increased resistance with newer strains of the dominant Omicron variant, these therapies were removed from the algorithm in April 2022.

**Results:**

A total of 139 children were treated with COVID-19 therapies based on having a significant underlying comorbidity meeting criteria [Table 1].

**Symptomatic, non-hypoxic COVID-19;** The majority (102, 73%) received treatment for symptomatic, non-hypoxic COVID-19 [Table 2]. Three drug-related adverse events were noted that resolved with treatment cessation; an infusion reaction with sotrovimab; taste disturbance with nirmatrelvir/ritonavir; and elevated liver enzymes with remdesivir [Table 3]. Six (2.9%) children re-presented within 90 days of therapy requiring hospitalisation [Table 4]; 4 (3.9%) with ongoing COVID-19 symptoms requiring monitoring and 2 (2%) with suspected secondary infections.

**Symptomatic, hypoxic COVID-19**; Thirty-seven (26.6 %) children were treated for symptomatic, hypoxic COVID-19 [Table 2]. Of these, 31 (83.8%) received dexamethasone; 9 (24.3%) remdesivir; 5 (13.5%) baricitinib; 5 (13.5%) tocilizumab, 1 (2.7%) sotrovimab and 1 (2.7%) anakinra. One death occurred, attributed to complications secondary to a bone marrow transplant and was unrelated to COVID-19 directed therapy. There were no other adverse effects. Early cessation of therapies prior to course completion was indicative of symptom resolution and discharge from hospital. Overall clinical course for symptomatic, hypoxic COVID-19 patients is outlined in Table 5. Hospitalisation within 90 days of receiving COVID-19 therapies was required for 4 (10.8%) patients; 3 (8.1%) with ongoing COVID-19 symptoms requiring monitoring and 1 (2.7%) with a suspected secondary infection [Table 4].

**Table 1:**
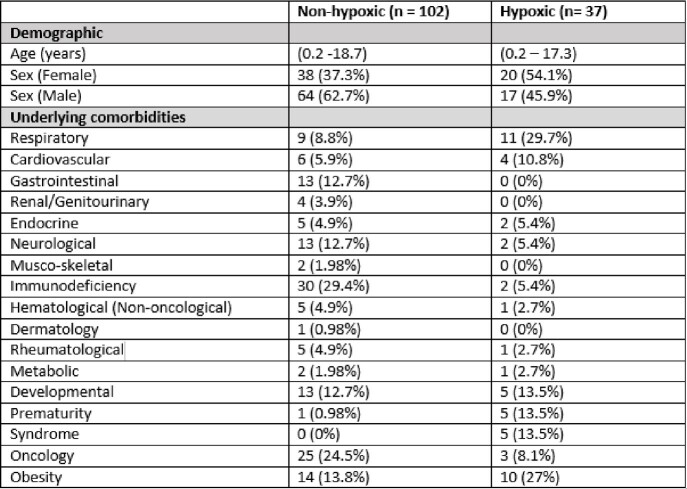
Underlying co-morbidities of patients treated with COVID-19 therapies over study period.

**Table 2:**
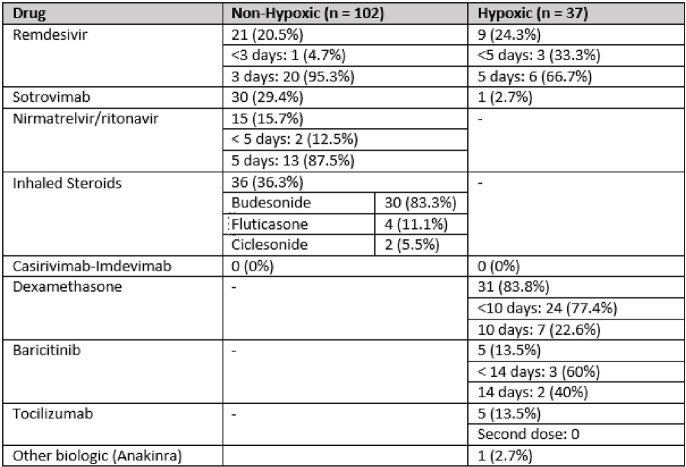
Treatments initiated during study period

**Table 3:**
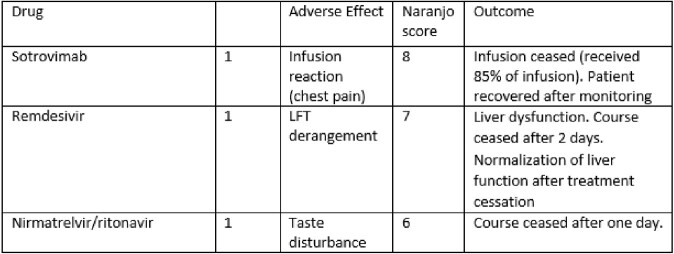
Adverse events during study period

**Conclusion:**

The use of directed COVID-19 therapies in high-risk paediatric patients were largely well-tolerated and side effects self-resolved upon treatment cessation.

**Table 4:**
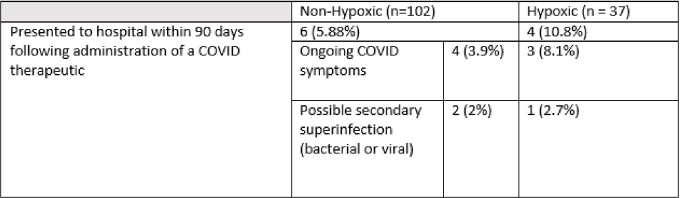
Hospitalisations within 90 days of receiving COVID-19 directed therapy

**Table 5:**
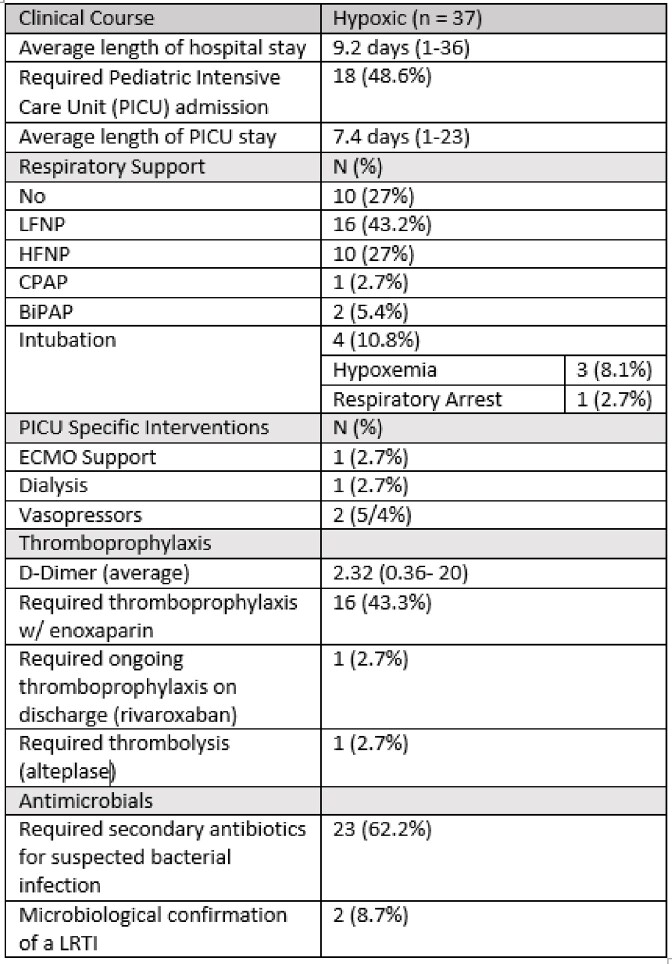
Clinical course of patients treated via the symptomatic, hypoxic COVID-19 pathway

**Disclosures:**

**All Authors**: No reported disclosures

